# Knowledge, attitudes and practice among physicians during the COVID-19 pandemic

**DOI:** 10.1590/1516-3180.2020.040320072020

**Published:** 2020-08-14

**Authors:** Viroj Wiwanitkit

**Affiliations:** I MD. Honorary professor, Dr DY Patil University, Pune, India.

Dear Editor,

I would like to share some ideas on the article “Evaluation of knowledge and attitudes among intensive care physicians during the COVID-19 pandemic: a cross-sectional survey”, which was published in the Sao Paulo Medical Journal.^1^ Erbas et al. concluded that “*For intensive care treatment of ­COVID-19 patients, many factors require management, and clinicians’ experience is guiding future processes*”.^1^

Since COVID-19 is a new emerging disease, the data available for effective diagnostic and therapeutic management are limited. When a disease first occurs in a country, there is no doubt that practitioners usually only have limited knowledge. For example, in Thailand, the second country in which COVID-19 occurred,^2^ the knowledge of local practitioners was not good when the disease first occurred.^3^

Therefore, the key important thing is medical education for practitioners. During a crisis, the data available changes rapidly and there is usually a problem in communication. Information technology (IT) may play a role, but its availability remains limited in remote areas. Additionally, because of the high influx of patients, practitioners might not have any time for education. A good plan for medical education for practitioners who have to care for these patients is necessary.
